# Medical Items

**Published:** 1892-11

**Authors:** 


### *MEDICAL ITEMS.*

THE office of the JOURNAL has been removed to room 330, Equitable building.

DR .M. B. HUTCHINS has moved his office to the Equitable building, room 330.

AN aspiring Chicago lecturer on Children's Diseases states that he expects soon to be made full professor of pæderasty.

A NEW local anæsthetic has been discovered by Giesel, which he calls *tropsin.* It is produced from the leaves of the cocoa plant, but has no relation to cocaine. For local anæsthesia, especially in eye surgery, it is said to be preferable to the latter.

SIR JOSEPH LISTER retires from his post as Lecturer on Surgery at King's College, London, through the operation of a rule which provides for the resignation of teachers at the age of sixty-five. By special courtesy he remains surgeon to the hospital for another year.

---

FROM private correspondence we learn that there is a good opening at Darien, Ga., "for a young physician who will keep in connection with his practice a small drug stock." Any worthy young man who will like to build up a country practice will do well to investigate.

---

THE Southern Surgical and Gynecological Association will hold its fifth annual meeting in the city of Louisville, Tuesday, Wednesday and Thursday, November 15, 16 and 17, under the presidency of Dr. J. McF. Gaston, of Atlanta. Members of the medical profession are most cordially invited to attend.

---

WE congratulate our friend, Dr. Chas. M. Blackford, of Lynchburg, Va., on receiving Dr. McGuire's prize of one hundred dollars for the best article on tetanus presented at the Virginia Medical Society, in September. The results of his investigations are thus summed up (*N. Y. Med. Jour.*): 1. Tetanus consists essentially of a tonic spasm of certain muscles which have been thrown into a state of physiological tetanus. 2. This is caused by abnormal irritability of the reflex centers of the medulla and cord. 3. This hyperæsthesia is the result of the physiological action of certain ptomaines, or alkaloids of decomposition, formed in the wound and absorbed therefrom. 4. The ptomaines are the result of the growth of a specific bacterium called the bacillus of tetanus or the bacillus of Nicolaier, and only by it. Therefore, 5, tetanus is a toxic disease, caused by the infection of a wound by this specific bacterium or its products.

